# The Chemical and Pharmacological Properties of Mitragynine and Its Diastereomers: An Insight Review

**DOI:** 10.3389/fphar.2022.805986

**Published:** 2022-02-24

**Authors:** Thiruventhan Karunakaran, Kok Zhuo Ngew, Ahmad Alif Danial Zailan, Vivien Yi Mian Jong, Mohamad Hafizi Abu Bakar

**Affiliations:** ^1^ Centre for Drug Research, Universiti Sains Malaysia, Pulau Pinang, Malaysia; ^2^ School of Chemical Sciences, Universiti Sains Malaysia, Pulau Pinang, Malaysia; ^3^ Centre of Applied Science Studies, Universiti Teknologi MARA, Kuching, Malaysia; ^4^ Bioprocess Technology Division, School of Industrial Technology, Universiti Sains Malaysia, Pulau Pinang, Malaysia

**Keywords:** mitragynine, diastereomers, indole alkaloids, analgesic, opioid receptor

## Abstract

Mitragynine, is a naturally occurring indole alkaloid that can be isolated from the leaves of a psychoactive medicinal plant. *Mitragyna speciosa*, also known as kratom, is found to possess promising analgesic effects on mediating the opioid receptors such as *µ* (MOR), *δ* (DOR), and *κ* (KOR). This alkaloid has therapeutic potential for pain management as it has limited adverse effect compared to a classical opioid, morphine. Mitragynine is frequently regarded to behave like an opioid but possesses milder withdrawal symptoms. The use of this alkaloid as the source of an analgesic candidate has been proven through comprehensive preclinical and clinical studies. The present data have shown that mitragynine is able to bind to opioid receptors, particularly MOR, to exhibit the analgesic effect. Moreover, the chemical and pharmacological aspects of mitragynine and its diastereomers, speciogynine, speciociliatine, and mitraciliatine, are discussed. It is interesting to know how the difference in stereochemical configuration could lead to the difference in the bioactivity of the respective compounds. Hence, in this review, the updated pharmacological and toxicological properties of mitragynine and its diastereomers are discussed to render a comprehensive understanding of the pharmacological properties of mitragynine and its diastereomers based on their structure–activity relationship study.

## Introduction

**FIGURE 1:**
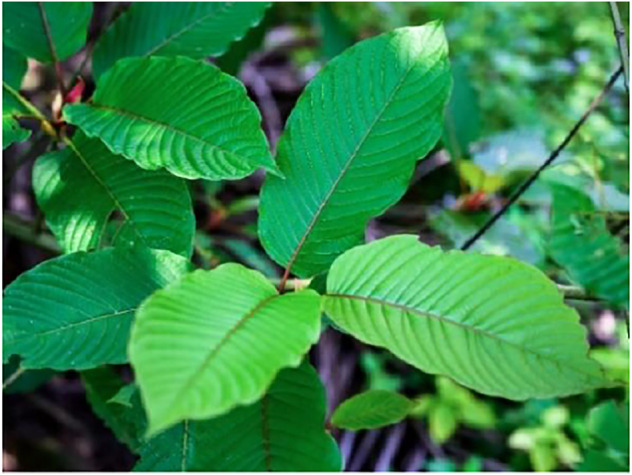
*M. speciosa* leaves.

Mitragynine **(1)** is an interesting natural product in the class of alkaloids that can be primarily isolated from the leaves of a medicinal plant, known as *Mitragyna speciosa* Korth (Gogineni et al., 2015). *M. speciosa* (Figure 1) is an indigenous and popularly cultivated plant from the Rubiaceae (coffee) family that grows in Southeast Asia, especially Malaysia and Thailand (Brown et al., 2017). In Malaysia, it is called ketum or biak-biak, while in Thailand, the plant is commonly known as kratom (Papsun et al., 2019; Chakraborty and Majumdar, 2020; Goh et al., 2021). Locals from southern Thailand and northern Malaysian Peninsular traditionally consumed the aqueous decoction for its distinctive medicinal properties to treat a variety of ailments such as diarrhea, muscle pain, and hypertension (Ilmie et al., 2015; Meireles et al., 2019).

The mitragynine **(1)** content in the leaves of *M. speciosa* varies considerably and is affected by geographical and climate conditions (Boffa et al., 2018). For example, Takayama (2004) found that mitragynine **(1)** contained about 12% mitragynine content in *M. speciosa* cultivated in Malaysia; meanwhile, Thailand’s *M. speciosa* possessed a much higher concentration of 66% mitragynine content. However, a recent finding by Goh et al. (2021) found that the mitragynine content in the Malaysian *M. speciosa* variant to be between 6.53% and 7.19%. The variation in mitragynine content could be attributed to several factors that may intrinsically affect the main constituent content, such as different chemotypes, climate, environmental pressures, and soil types (Chear et al., 2021).

Apart from mitragynine **(1)**, other interesting alkaloids are also present in considerable amounts in *M. speciosa*, especially in leaves. To date, about 54 alkaloids have been successfully isolated and identified from this species, which begin from the isolation of indole-alkaloid, mitragynine **(1),** by Ellen Field in the year 1921 (Flores-Bocanegra et al., 2020). Subsequently, other alkaloids from *M. speciosa* were discovered. Mitragynine’s **(1)** diastereomers, speciogynine **(2)**, speciociliatine **(3),** and mitraciliatine **(4)** were some of the prominent indole alkaloids that were found in *M. speciosa*. The biosynthesis of mitragynine **(1)** and its diastereomers is a complex system that involves various enzymatic steps in forming the respective phytoconstituents. Jumali et al. (2011) and Chear et al. (2021) have proposed putatively possible biosynthetic pathways where these phytoalkaloids were synthesized *via* a typical indole alkaloid pathway, starting from the shikimic acid pathway together with the methyl-erythritol phosphate pathway (MEP). Corresponding to mitragynine **(1)**, the content of the analogues of mitragynine **(1)** also varies significantly based on regional varieties and the maturity of the plant (Pearson et al., 2018). For instance, the leaves of matured *M. speciosa* plant contain the main alkaloid mitragynine **(1)**, its diastereomers speciogynine **(2)**, and speciociliatine **(3)**, as well as paynantheine, while the leaves of the younger plants contain mitragynine **(1)**, speciogynine **(2)**, speciociliatine **(3)**, and some small amounts of the mitragynine’s **(1)** diastereomer, mitraciliatine **(4)** (Philipp et al., 2010; Boffa et al., 2018).

Although this plant has been widely consumed due to its medicinal properties such as analgesic effect, mitragynine **(1)** have been found to be the major alkaloid in this traditional herb despite having opium-like effect at higher doses (Wilson et al., 2020). The local agriculture community commonly consumed it as a stimulant to increase endurance and counteract fatigue while working under the hot sun (Veltri and Grundmann, 2019; Ya et al., 2019). Several studies indicate that mitragynine **(1)** exhibits similar effects as cocaine (coca-like effects) and opium on the human body (Abdullah and Ismail, 2018; Wilson et al., 2020).

Moreover, mitragynine **(1)** and its diastereomers were reported to be widely metabolized by phase I and phase II metabolic enzymes into relevant metabolites based on animal and human studies (Kruegel et al., 2019; Chakraborty et al., 2021a). Metabolism of mitragynine **(1)** was first elucidated by Philipp et al. (2009). Phase I metabolism of mitragynine **(1)** and its diastereomers involved the hydrolysis of methyl ester of the propenoic acid at C-16 whereas *O-*demethylation of the methoxy group positioned at C-9 and C-17, respectively followed by oxidation or reduction reactions to form carboxylic acid or alcohol (Hanapi et al., 2021). In the human liver microsomes (HLM) system, 7-hydroxymitragynine and 9-*O*-demethylmitragynine were discovered as the most prevalent metabolites of mitragynine **(1)** (Basiliere and Kerrigan, 2020).

## Chemistry

**FIGURE 2:**
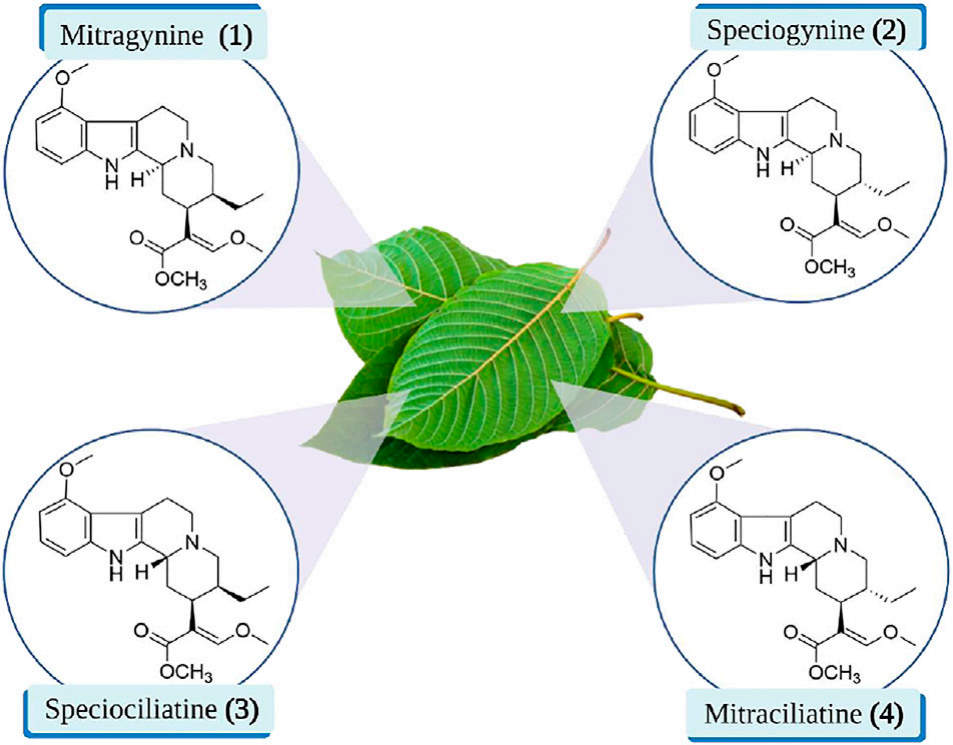
Chemical structures of mitragynine **(1)** and its diastereomers.

Mitragynine **(1)** is a corynanthe-type monoterpene indole alkaloid. Mitragynine congeners especially its diastereomers were found to be present in the leaves of *M. speciosa* which are speciogynine **(2)**, speciociliatine **(3)**, and mitraciliatine **(4)** (Raffa, 2015). Since the diastereomeric phytoconstituents are congeners of mitragynine that have the tetracyclic indole alkaloid core structure, these compounds can be distinguished through the structural configuration at certain important positions (Figure 2) (Ellis et al., 2020). Based on the chemical structure of these compounds, the difference in the configurational positioning at C-3, C-15, and C-20 results in the occurrence of mitragynine **(1)**, speciogynine **(2)**, speciociliatine **(3)**, and mitraciliatine **(4)**, respectively. The summary of absolute configurations (*R* or *S*) at the positioning of C-3, C-15, and C-20 of the respective compounds **1**–**4** are shown in Table 1.

**TABLE 1:** The absolute configurations of mitragynine **(1)** and its diastereomers.

Compound	C-3	C-15	C-20
Mitragynine **(1)**	*S*	*S*	*S*
Speciogynine **(2)**	*S*	*S*	*R*
Speciociliatine **(3)**	*R*	*S*	*S*
Mitraciliatine **(4)**	*R*	*S*	*R*

Despite the discovery of over 54 alkaloids from the leaves of *M. speciosa*, most research focused on the major constituent in the plant, which is mitragynine **(1)** (Flores-Bocanegra et al., 2020). Historically, mitragynine **(1)** was first isolated by Ellen Field in 1921, and later, the structure was completely characterized and elucidated by Beckett and Zacharias in 1965 (Gogineni et al., 2015). Furthermore, the diastereomers of mitragynine **(1),** speciogynine **(2)**, speciociliatine **(3)**, and mitraciliatine **(4)** were also reported to be isolated from the leaves of *M. speciosa*.

Based on the stereochemical configuration on the structure of mitragynine **(1)** and its diastereomers at position C-3, mitragynine **(1)** and speciogynine **(2)** possesses a flat trans-quinolizidine conformation in the rings of C and D as compared with a cis-quinolizine conformation in speciociliatine **(3)** (Takayama, 2004). In addition, speciociliatine **(3)** has a different spatial arrangement in comparison with mitragynine **(1)**, where both structures can be distinguished by a switch in the configuration from *R* [speciociliatine **(3)**] to *S* (mitragynine **(1)**) of the hydrogen moiety positioned at C-3. This configurational inversion from *R* to *S* will induce significant spatial change in the core skeleton (ring C and D) of speciociliatine **(3)**, where it will enhance its molecular volume while the inversion to mitragynine **(1)** will cause the *β*-methoxy acrylate moiety in the compound to adopt an axial position (Berthold et al., 2021).

### Isolation of Mitragynine (1) and its Diastereomers

The first isolation of mitragynine **(1)** was reported by Field, a Scottish chemist in 1921 (Kruegel and Grundmann, 2018). Subsequently, Beckett et al. (1965) established the chemical structure of mitragynine **(1)** while the absolute configuration of the compound was later confirmed by Zacharias et al. (1965) using the X-ray crystallographic method (Gogineni et al., 2015; Flores-Bocanegra et al., 2020). Raffa (2015) reported that decades later, the diastereomers of mitragynine **(1)**, speciogynine **(2)**, and speciociliatine **(3)** were discovered and isolated by Beckett et al. (1965) and Shellard et al. (1978). Another diastereomer, mitraciliatine **(4),** was also reported from the leaves of *M. speciosa* (Flores-Bocanegra et al., 2020). The increased interest in the alkaloids of *M. speciosa* by natural products and medicinal chemists had led to an increasing amount of research conducted in isolating other phytoconstituents by using various chromatographic techniques. However, there are issues regarding the purity of the alkaloids isolated from *M. speciosa* due to the difficulty in separating and isolating isomeric alkaloids. Goh et al. (2021) found a fast and rapid method in the isolation of mitragynine **(1)** with a peak purity of 98%, which was affirmed using HPLC analysis. Meanwhile, Chear et al. (2021) reported on the isolation of speciogynine **(2)** and speciociliatine **(3)** with high purity (≥98%) using column chromatographic techniques. These studies provide an understanding in solving the purity issue of the alkaloid drugs. It also prompted researchers to develop a set of guidelines to ensure that the purity (≥95%) is within the required guidelines as it is vital for preclinical and clinical studies.

### Characterization of Mitragynine (1) and Its Diastereomers

**FIGURE 3:**
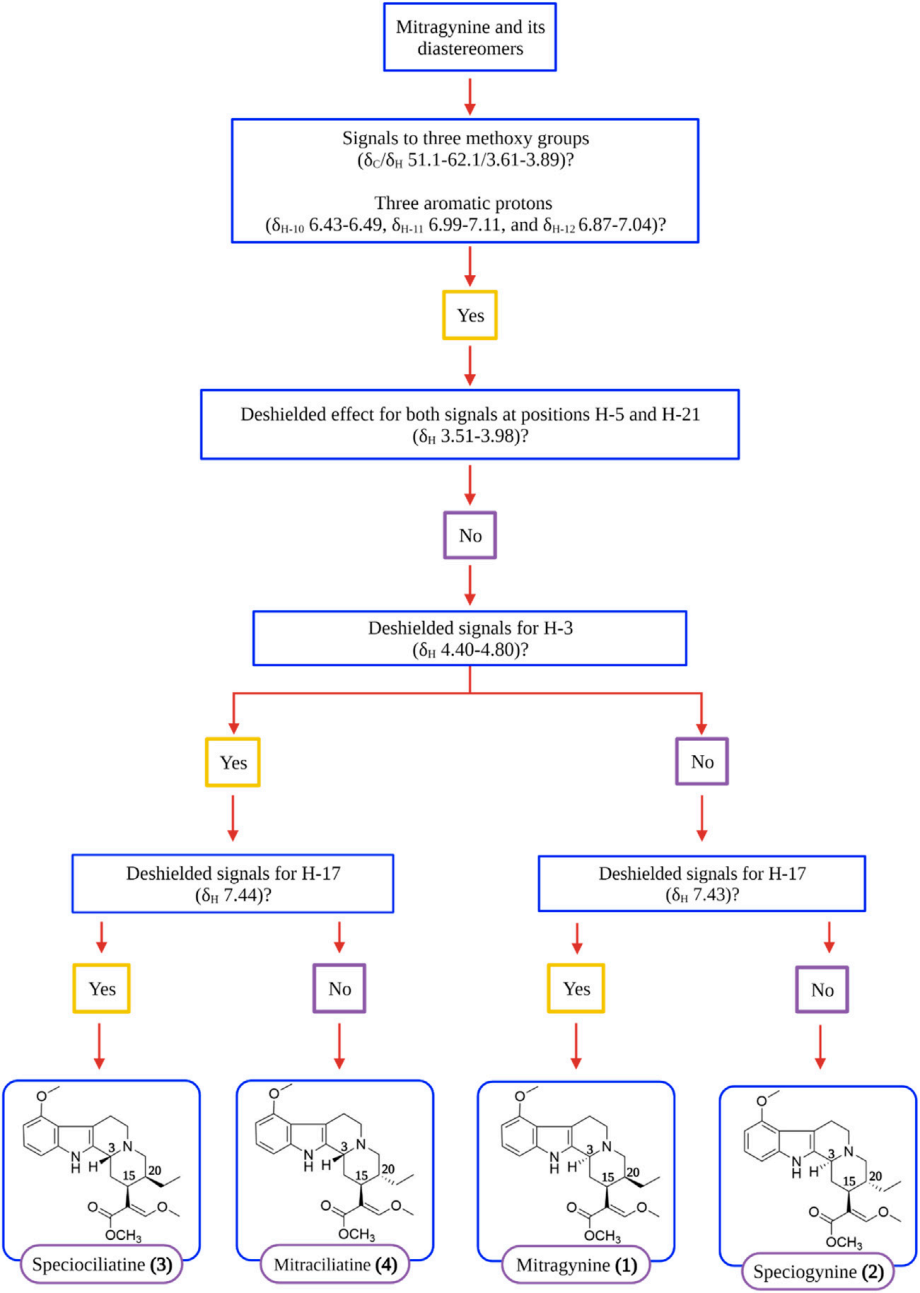
Decision tree on distinguishing mitragynine **(1)** and its diastereomers by main NMR signals (Flores-Bocanegra et al., 2020).

Complete characterization and elucidation of mitragynine **(1)** and its diastereomers were reported recently by Flores-Bocanegra et al. (2020) and Chear et al. (2021) using nuclear magnetic resonance (NMR) and mass spectrometry (MS) analyses. Flores-Bocanegra et al. (2020) devised a simple and comprehensive decision tree to distinguish the indole and oxindole alkaloids discovered from *M. speciosa* through the identification of important chemical shifts such as ^1^H and ^13^C NMR signals. Hence, Figure 3 showed the simplified version of the decision tree chart as adapted from Flores-Bocanegra et al. (2020) where the flow of decision for the identification of mitragynine **(1)** and its diastereomers, speciogynine **(2)**, speciociliatine **(3)**, and mitraciliatine **(4)**, are comprehensively depicted. The references for spectral data of mitragynine **(1)** and its diastereomers are tabulated in Table 2.

**TABLE 2:** Reference spectral data for mitragynine **(1)** and its diastereomers.

Compound	Spectral data
Mitragynine (1)	^1^H (600 MHz & 400 MHz), ^13^C (150 MHz & 100 MHz) NMR and HRESIMS data (Chear et al., 2021 and Flores-Bocanegra et al., 2020)
Speciogynine (2)	
Speciociliatine (3)	
Mitraciliatine (4)	^1^H (400 MHz), ^13^C (100 MHz) NMR and HRESIMS data (Flores-Bocanegra et al., 2020)

## Pharmacology/Structure Activity Relationship Study (SARs)

**FIGURE 4:**
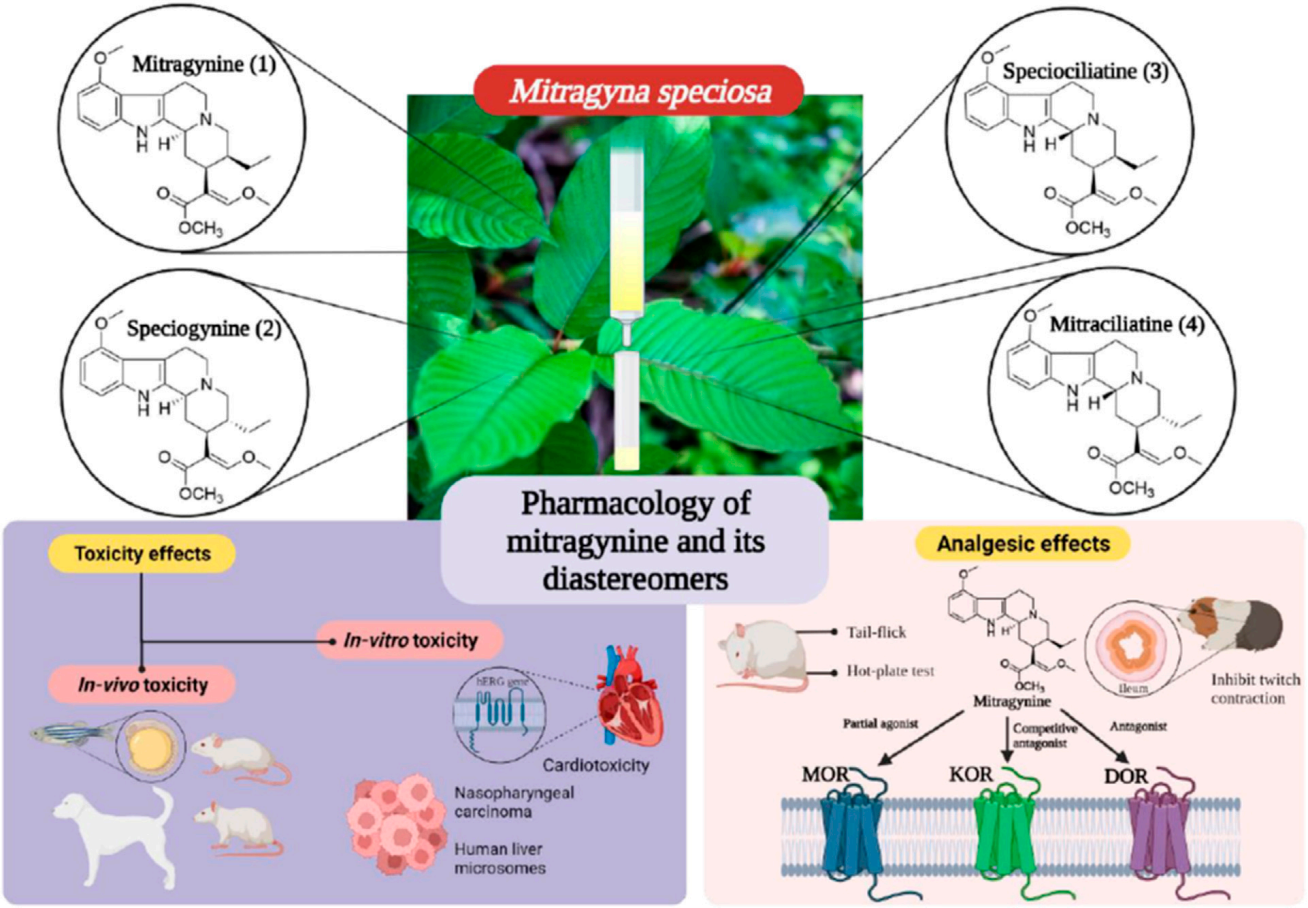
Selected pharmacological activities on mitragynine **(1)** and its diastereomers.

The pharmacological activity of the alkaloids from *M. speciosa* has been extensively researched, focusing on the analgesic potency of the primary indole alkaloid, mitragynine **(1)**. Isomeric indole alkaloids such as mitragynine **(1)**, speciogynine **(2)**, and speciociliatine **(3)** were assessed through *in vivo* and *in vitro* approaches for their pharmacological properties, especially in assessing their analgesic and toxicological properties. Takayama (2004) accumulated evidence implicating the opioid receptor system as the primary mediator of the central nervous system effects displayed by these isomeric phytoalkaloids. To the best of our knowledge, we found very limited pharmacological evidence on mitraciliatine **(4)**. The pharmacological properties of these alkaloids are shown below. Selected pharmacological activities on mitragynine and its diastereomers are summarized in Figure 4.

Macko et al. (1972) first reported the pharmacological studies of mitragynine **(1)**. The analgesic potency of mitragynine **(1)** has been studied mostly through tail-flick and hot plate tests. It was found to induce antinociception in the brain. It was also reported that the antinociceptive action of mitragynine in mice was at least partly involved in the supraspinal opioid systems (Matsumoto et al., 1996). Currently, detailed pharmacology studies are being conducted on mitragynine **(1)** and its diastereomers to understand their mechanism of action and their structure–activity relationship in pain management.

**FIGURE 5:**
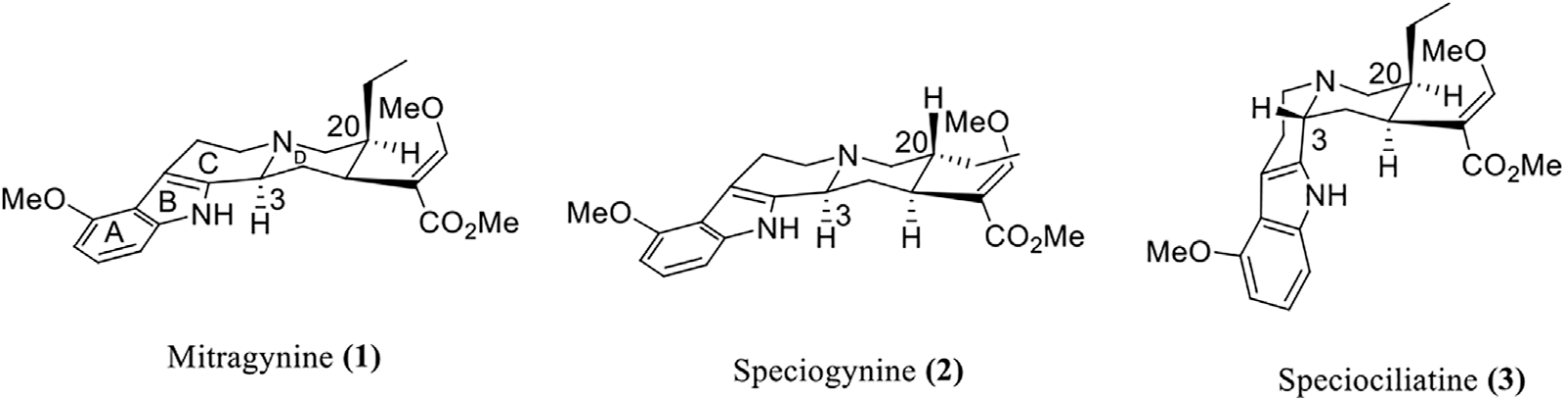
Stereochemical conformation of mitragynine **(1)**, speciogynine **(2)**, and speciociliatine **(3)**.

A preliminary study conducted by Takayama et al. (2002) on the biological activities of *M. speciosa* crude leave extract with several bioactive alkaloids showed promising results. The first noteworthy result was shown through the ability of mitragynine **(1)** in inhibiting the twitch contraction of guinea pig ileum, which is induced by electrical stimulation. It was reported that the opioid agonistic activities of mitragynine **(1)** and speciociliatine **(3)** were evaluated based on their ability to inhibit contraction that stimulates the guinea pig ileum, which is reversed by a classical antagonist, naloxone. Mitragynine **(1)**, as portrayed in Figure 5 below, possesses a unique flat trans-quinolizidine conformation in the C/D ring junction, while speciociliatine **(3)**, a C-3 diastereomer of mitragynine **(1)**, assumes a folded cis-quinolizidine conformation.

Based on the opioid agonistic activities of mitragynine **(1)** and speciociliatine **(3)** in electrically stimulated guinea-pig ileum (Table 3), the potency of speciociliatine **(3)** toward the mu opioid receptor (MOR) was shown to be 13 times weaker than mitragynine **(1)**. The pD_2_, also known as pEC_50_, is the negative logarithm to base 10 of the EC_50_ of an agonist. The pD_2_ value indicates the potency of an agonist but not its efficacy. Based on the pD_2_ value observed in Table 3, the performance of mitragynine (pD_2_ = 6.95 ± 0.12) as an opioid agonist was weaker compared to morphine (pD_2_ = 7.17 ± 0.05). This implies that the flat trans-quinolizine conformation form in mitragynine **(1)** was a more efficient conformation to exhibit the opioid activity than folded cis-quinolizidine.

**TABLE 3:** Opioid Agonistic Activity of Mitragynine **(1)**, Speciociliatine **(3)**, and Morphine in Electrically Stimulated Guinea-Pig Ileum Preparation.

Compound	Structure	^a^pD_2_ value	^b^Relative potency (%)	^c^Relative inhibitory activity (%)
Mitragynine **(1)**	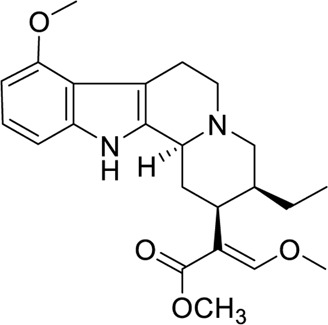	6.95 ± 0.12	26	95
Speciociliatine **(3)**	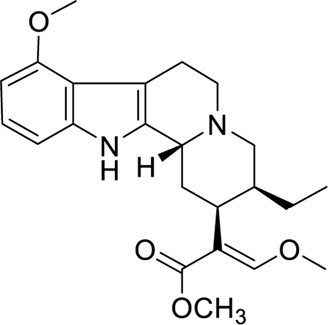	5.40 ± 0.07	2	101
Morphine	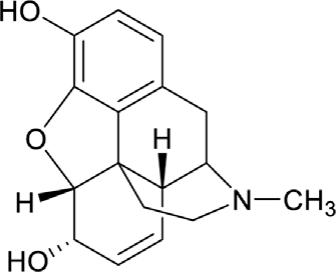	7.17 ± 0.05	100	100

a pD_2_ values indicate the potency of agonist, the higher pD_2_ reflects higher potency of the agonist.

b Relative potency is shown as a percentage of the pD_2_ value of the compound against that of morphine.

c Relative inhibitory activity reflects to the intrinsic activity on opioid receptors, is shown as a percentage of the maximum inhibition by compounds against that by morphine. All data points represent mean ± SEM (µM) of *n* ≥ 3.

Based on the *in vitro* studies conducted by Takayama et al. (2002), mitragynine **(1)** was reported to have a significant binding affinity on the opioid receptor. This study was conducted on the guinea pig brain homogenates. There is a total of three ligand subtypes that were used to assess the binding affinities of the compound mitragynine **(1)** and on the opioid receptor subtypes. They were MOR-selective ligand DAMGO ([D-Ala^2^, *N*-MePhe^4^, Gly-ol]-enkephalin), the DOR-selective ligand DPDPE (D-Pen^2^-D-Pen^5^-enkephalin), and the KOR-selective ligand U-69,593 (N-methyl-2-phenyl-N-[(5*R*,7*S*,8*S*)-7-(pyrrolidin-1-yl)-1-oxaspiro [4.5] dec-8-yl] acetamide). From this study, it was shown that the affinity of mitragynine **(1)** at MOR, DOR, and KOR is 7.2, 60, and >1,000 nM, respectively. It also displayed a nearly 10-fold selectivity for MOR over DOR sites and greater than 1,000-fold selectivity for MOR over KOR.

Kruegel et al. (2016) studied the activity of mitragynine **(1)** and its other analogues speciogynine **(2)** and speciociliatine **(3)**. The study was conducted on HEK cell lines expressing the human opioid receptor. The results indicate that mitragynine **(1)** acts as a partial agonist at MOR; meanwhile, speciogynine **(2)** and speciociliatine **(3)** were both weak antagonists at MOR. Additionally, it was also found that mitragynine **(1)** and its diastereomers bind significantly at MOR than KOR and DOR (Kruegel et al., 2016; Zhou et al., 2021). A deeper view on the binding affinities of mitragynine **(1)** and its diastereomer showed that mitragynine **(1)** has the highest binding affinities, followed by speciociliatine **(3)** and speciogynine **(2)** (Obeng et al., 2021). Even though mitragynine **(1)** and its diastereomer bind actively at MOR, its activity at the receptor is not identical, as shown in Table 4.

**TABLE 4:** Binding Affinities of Mitragynine **(1)** and its Diastereomers at Human Opioid Receptor (Obeng et al., 2021).

Compound	K_i_ ± SEM (µM)^a^		
	hMOR (µ)	hKOR (κ)	hDOR (δ)
Mitragynine **(1)**	0.233 ± 0.048	0.772 ± 0.207	>10
Speciogynine **(2)**	0.728 ± 0.061	3.200 ± 0.360	>10
Speciociliatine **(3)**	0.560 ± 0.168	0.329 ± 0.112	>10

a All data points present mean ± SEM (*µ*M) of *n* ≥ 3.

Based on Table 5 above, mitragynine **(1)** act as a partial agonist at MOR with a maximal efficacy of 34%. However, at KOR and DOR, the functional activity of mitragynine **(1)** alkaloid converts from partial agonist to antagonist at lower potency. Additionally, the diastereomers of mitragynine **(1)**, speciogynine **(2)**, and speciociliatine **(3)** showed null measurable agonist activity at all the opioid receptors and only revealed a weak antagonist effect. By comparing mitragynine **(1)** and speciogynine **(2)**, the ethyl group at position 20 on ring D shows a crucial point as the epimerization of this group is able to switch the agonistic activity to antagonist activity at MOR. The modification of the configuration of the ethyl group also reduced the binding affinity.

**TABLE 5:** The Functional Activity of *M. speciosa* Alkaloids at Human Opioid Receptors in G protein BRET Assays (Kruegel et al., 2016).

Compound	EC_50_ ± SEM (E_max_)^a^ or [ IC_50_ ± SEM (pA_2_)]^b^		
	hMOR	hKOR	hDOR
Mitragynine **(1)**	0.339 ± 0.178 (34%) (partial agonist)	8.5 ± 7.6 (1.4) (competitive antagonist)	>10 (antagonist)
Speciogynine **(2)**	5.7 ± 2.8 (weak antagonist effect)	>10 (weak antagonist effect)	>10 (weak antagonist effect)
Speciociliatine **(3)**	4.2 ± 1.6 (weak antagonist effect)	>10 (weak antagonist effect)	>10 (weak antagonist effect)

a EC_50_ values indicate the agonist activity, (E max) relative to DAMGO, in parentheses.

b IC_50_ values indicate the inhibition of a reference agonist, (pA_2_) determined from Schild analysis in parentheses.

All data points represent mean ± SEM (µM) of *n* ≥ 3.

Obeng et al. (2020) carried out a pharmacological investigation on mitragynine **(1)** and speciociliatine **(3)** to evaluate its opioid binding affinities (Table 6). According to the results obtained in Table 7, the binding affinities of speciociliatine **(3)** (K_i_ (MOR): 116 ± 36 nM, K_i_ (KOR): 54.5 ± 4.4 nM) at both opioid receptors are higher than mitragynine **(1)** (K_i_ (MOR): 198 ± 30 nM, K_i_ (KOR): 161 ± 10 nM). Berthold et al. (2021) also found that the binding affinity of speciociliatine **(3)** at MOR and KOR was 3.0- and 1.7-fold higher than that of mitragynine **(1)**. Based on the two studies, it is suggested that the conversion of the configuration at position 3 from *S* [mitragynine **(1)**] to *R* [speciociliatine **(3)**] causes a significant change in terms of the binding affinities. This switch in conformation speciociliatine **(3)** will cause the molecular volume of speciociliatine **(3)** to have a larger space to bind and interact with the active sites of the opioid receptor and increase its binding affinities compared to mitragynine **(1)**. Based on molecular docking studies, the acrylate moiety will affect the interaction with the key residue, which plays an important role in binding to the opioid receptors. However, these results were contrary to what was reported by Kruegel et al. (2016), who found that speciociliatine **(3)** had no significant agonist activity at all the human opioid receptors and acted as a weak antagonist. The difference might be due to different assay types used to evaluate speciociliatine **(3)**. This result was confirmed by a study by Nickolls et al. (2011) that different types of assays used to evaluate the targeted compound will show different agonistic effects.

**TABLE 6:** Screening of mitragynine **(1)** and speciociliatine **(3)** at opioid receptor (Obeng et al., 2020).

Binding site	Percent displacement of bound radioligand			
	Mitragynine (1)		Speciociliatine (3)	
	100 nM	10,000 nM	100 nM	10,000 nM
MOR	29.0	93.7	64.7	98.0
KOR	25.2	88.3	61.9	98.5
DOR	0.4	18.3	0.6	69.2
Nociceptin/Orphanin FQ peptide (NOP)	4.3	40.8	−14.9	31.8

**TABLE 7:** Binding affinities of mitragynine **(1)** and speciociliatine **(3)** to opioid receptor and subtype selectivity (Obeng et al., 2020).

Compound	DOP K_i_ ± SEM (nM)	KOP K_i_ ± SEM (nM)	MOP K_i_ ± SEM (nM)
DAMGO	ND	ND	0.41 ± 0.04
DPDPE	1.32 ± 0.004	ND	ND
U50488	ND	0.300 ± 0.002	ND
Mitragynine **(1)**	ND	198 ± 30	161 ± 10
Speciociliatine **(3)**	ND	116 ± 36	54.5 ± 4.4

The ethyl group in ring D is extremely crucial in predicting the binding affinities at the opioid receptor. The ethyl group will act as a hydrophobic group that interacts with the receptor. For the binding affinities of the structure without the ethyl group, the binding affinities are diminished drastically as compared to mitragynine **(1)**. All in all, the number of different stereochemical configurations in the diastereomers can retain to bind at MOR. Meanwhile, absolute stereochemistry is found to be crucial in agonistic activity in the opioid receptor.

In a recent report by Chakraborty et al. (2021b) on the opioid receptor function of mitraciliatine **(4)**, pharmacological evidence was reported on this phytoalkaloid. In this study, mitraciliatine **(4)** (K_i_ (MOR): 135.1 ± 7.7 nM) portrayed partial opioid agonism and identified it as structurally unique natural products with safer, MOR-dependent antinociception. Mitragynine **(1)** [K_i_ (KOR): 231 ± 21 nM] was reported to have weak KOR antagonism in contrast with mitraciliatine **(4)** (K_i_ (KOR): 101.2 ± 2.3 nM, K_i_ (MOR): 6.52 ± 0.06 nM), which showed KOR full agonism and MOR partial agonism at both human and mouse receptors. Mitraciliatine **(4)** (E_max_ (KOR): 104%) showed robust *β* arrestin-2 recruitment at KOR, while it does not recruit *β* arrestin-2 at MOR. Based on this, it appears that mitraciliatine **(4)** (Ki (KOR): 73 nM)/(Ki (MOR): 304 nM) has a higher receptor selectivity for opioids over adrenergic receptors with fewer off-target interactions. The activity portrayed might be due to its stereochemical configuration at C-3 [*S* in mitragynine **(1)** and *R* for mitraciliatine **(4)**] and C-20 [*S* in mitragynine **(1)** and *R* for mitraciliatine **(4)**], which plays a vital role in the SAR of the respective phytoalkaloids. Chakraborty et al. (2021c) also performed an investigation on mitragynine **(1)** and its synthesized analogues, focusing on the C-9 position in the scaffold of mitragynine **(1)**. The three synthesized analogues, 9-3′-furanyl mitragynine, 9-phenyl mitragynine, and 9-methyl mitragynine, demonstrated partial agonism toward G-protein and arresting signals mediated by MORs. The synthesized analogues exhibit moderate activity and potency (EC_50_ > 50 nM) in cAMP assays and poor *β*-arrestin2 recruitment (E_max_ < 20%) at MOR. The semisynthetic modifications of mitragynine **(1)** at C-9 position using moieties such as 3′-furanyl, phenyl, and methyl do not enhance the potency toward the tested activities.

A previous study conducted by Matsumoto et al. (1997) had affirmed that mitragynine **(1)** displayed a suppressive effect on the central serotonin neurotransmission system. In mice, the suppression of 5-HT_2A_ agonist (5-methoxy-N, N-dimethyltryptamine)-induced head twitch response was observed due to the effect from the pre-treatment with mitragynine **(1)**, which showed that the principal kratom alkaloid acts as a competitive antagonist in blocking the stimulation of the 5-HT_2A_ receptor (Hanapi et al., 2021). León et al. (2021) investigated the *in vitro* and *in vivo* activity of kratom alkaloids, especially mitragynine **(1)**, speciogynine **(2)**, and speciociliatine **(3)** at serotonin receptors (5-HTRs). Surprisingly, speciogynine **(2)** portrayed a high affinity toward 5-HT_1A_Rs and 5-HT_2B_Rs, in contrast with its major diastereomer, mitragynine **(1)**. Speciogynine **(2)** exhibited antinociceptive properties in rats *via* an opioid receptor-independent mechanism. Since mitragynine **(1)** (20*S*) and speciogynine **(2)** (20*R*) are diastereomers that differ in C-20 position, the structural difference of the *β*-methoxyacrylate group in both diastereomers might cause the difference in the potency toward the tested activity (León et al., 2021).

### Toxicity of Mitragynine (1) and Its Diastereomers

#### *In vitro* Toxicity

Several cytotoxic studies of mitragynine **(1)** and its diastereomers were conducted toward selected cancerous and non-cancerous cell lines. Lu et al. (2014) provided the first scientific evidence on cardiotoxicity of mitragynine **(1)** and its diastereomers. Based on the report, mitragynine **(1)** administered at 10 µM showed significant cardiotoxicity by inhibiting the human ether-à-go-go-related gene (hERG) current. Meanwhile, mitragynine **(1)** (10 µM) also prolongs action potential duration (APD) and induces arrhythmia. Moreover, mitragynine **(1)**, speciogynine **(2)**, and speciociliatine **(3)** were dosage-dependently (0.1, 100 µM) suppressed I_Kr_ in hiPSC-CMs at 67%∼84% with IC_50_ ranging from 0.91 to 2.47 µM. The inhibition of hERG has been associated with favorable binding of drugs to open and inactivated states of hERG channels. The inward potassium currents are primarily active during phase 1 of the cardiac AP, while calcium channels are primarily active during phase 3. QT prolongation is primarily an issue arising after depolarizations.

In 2015, Saidin et al. (2015) reported on the cytotoxicity of mitragynine **(1)** tested against SH-SY5Y and MCL-5 cell lines, respectively. Mitragynine (**1**) exhibited a moderate cytotoxic effect against these reported cell lines with IC_50_ values of 75 and 80 μM, respectively. Oliveira et al. (2016) also reported on moderate cytotoxicity of mitragynine **(1)** evaluated against Caco-2 (42.5 μg/ml) and SH-SY5Y (42.6 μg/ml) cell lines. The moderate activity of mitragynine could be attributed to the absence of OH moiety in its tetracyclic monoterpenoid indole alkaloid nucleus (Rosales et al., 2020).

Kamble et al. (2020) reported on the inhibition of CYP 450 isoforms in human liver microsomes by *M. speciosa* alkaloids, mainly mitragynine **(1)**, and its diastereomers. Cytochrome P450 (CYP450) is a group of enzymes that play a predominant role in drug metabolism; therefore, an alteration in CYP450-mediated metabolism could result in drug interactions that include fatality. The findings demonstrated that mitragynine **(1)** (IC_50_: 2.2 µM) was a potent and relatively selective inhibitor toward CYP2D6, while it possessed moderate inhibition with IC_50_ 11.4 µM toward CYP3A4/5 (an isoform of CYP450). Additionally, speciogynine **(2)** (IC_50_: 19.5 µM) and speciociliatine **(3)** (IC_50_: 8 µM) displayed moderate inhibition toward CYP2C19. However, there was no activity shown on mitragynine **(1)**, speciogynine **(2)**, and speciociliatine **(3)** toward the other tested CYP450 isoforms. Mitragynine **(1)** and its diastereomers have a more planar three-dimensional structure where the indoloquinazoline moiety of these compounds was completely overlapped. The planar structure of these three compounds might reflect in the strong inhibition of CYP2D6 and CYP3A4/5.

Another *in vitro* toxicity study on mitragynine **(1)** and its diastereomers was reported. Mitragynine **(1)**, speciogynine **(2)**, and speciociliatine **(3)** possessed weak-to-moderate inhibition against nasopharyngeal carcinomas NPC/HK-1 and C666-1 cell lines. In the study, mitragynine **(1)** exhibited the highest inhibition against the growth of NPC/HK-1 cells, followed by speciociliatine **(3)**. The overall SARs study revealed that the *R* and *S* orientations at positions C-3 and C-20, respectively, are the key features that determine the cytotoxicity of mitragynine **(1)** and its diastereomers. Besides, speciociliatine **(3)** was shown to possess weaker cytotoxicity than mitragynine **(1)** due to the inversion of orientation from *R* to *S* at the C-3 position. However, the cytotoxicity of speciogynine **(2)** was shown to be abolished due to the *R* orientation at the C-20 position of the respective compound (Domnic et al., 2021).

Additionally, Goh et al. (2021) found that mitragynine **(1)** possessed higher IC_50_ values against two cell lines, HEK-293 kidney cell (IC_50_: 112.30 ± 17.59 µM) and HeLa Chang liver cell (IC_50_: 210.04 ± 0.80 µM), being compared to all of the tested ASE *M. speciosa* extracts. Mitragynine **(1)** showed moderate toxicity toward these cell lines, which suggested that mitragynine **(1)** could have selectively exhibited a cytotoxicity effect on both cancerous and non-cancerous cell lines.

#### *In vivo* Toxicity

Initial studies on the *in vivo* toxicity of mitragynine **(1)** on rats and dogs were documented in the year 1972. A single dosing of mitragynine **(1)** (806 mg/kg) produced no toxicity in rats, and multiple oral 50 mg/kg/day also showed no observable side effects. Subsequently, the daily dosage of 16 mg/kg and two additional days of oral 32 mg/kg in dogs also showed no side effects. However, at higher doses and longer exposures, primarily, blood dyscrasias were also observed (Macko et al., 1972).

Sabetghadam et al. (2013) reported a relatively safe consumption of lower to sub-chronic amounts of mitragynine **(1)** in rats (1–10 mg/kg) but detected signs of toxicity at higher doses (100 mg/kg) when histopathological, hematological, and biochemical effects of the liver, kidney, and brain were observed. The authors suggested that the use of mitragynine **(1)** in the dose range studied is generally safe as there have been no deaths reported and no significant differences in overall behavior.

Additionally, Damodaran et al. (2021) reported on the toxicity assessment of two diastereomeric alkaloids, mitragynine **(1)** and speciociliatine **(3)**, on the zebrafish embryo model. This study aimed to assess the possible toxicity effects exhibited by the two alkaloids on the zebrafish embryo model with different toxicity parameters, namely, mortality, hatching rate, heart rate, and morphological malformations. It showed that acute embryonic exposure to mitragynine **(1)** and speciociliatine **(3)** affected the survival, hatching, and body morphology of zebrafish embryos in a concentration- and time-dependent manner, which indicates that higher compound concentrations and longer exposure times affect the development of zebrafish embryo. In the mortality assessment of zebrafish embryos, the LC_50_ value of mitragynine **(1)** was 32.01 μg/ml at 96 hpf, while the estimated LC_50_ value of speciociliatine **(3)** was slightly higher at 79.86 μg/ml, indicating that speciociliatine **(3)** is relatively safer than mitragynine **(1)**. The data obtained on the hatching parameter showed that mitragynine **(1)** and speciociliatine **(3)** at concentrations of 25 and 50 μg/ml appeared to be associated with the delayed hatching process, whereas for the morphological malformation parameter, spinal curvature (scoliosis) was observed in mitragynine **(1)** (50 μg/ml)- and speciociliatine **(3)** (25 and 50 μg/ml)-exposed groups. It was concluded that mitragynine **(1)** and speciociliatine **(3)** (≥50 μg/ml) possessed certain undesirable effects on embryonic development by affecting the survival, hatching, and body morphology of zebrafish embryos, which relays to the potential risk of kratom intake during pregnancy on the development of the fetus. This is because the early embryo developmental process of zebrafish is similar to humans.

## Conclusion

In conclusion, the mitragynine **(1)** template and its structural information could render medicinal chemists an opportunity to develop a new analgesic that can be beneficial toward pain management and treatment. It is vital for chemists and pharmacologists to determine its maximum analgesic potency as well as alter the opioid-induced side effects through detailed preclinical and clinical studies. The alkaloidal chemistry, especially focusing on the functional activity of mitragynine **(1)** and its diastereomers toward opioid receptors such as MOR, needs to be investigated in detail. The pharmacokinetic properties of the phytoalkaloids in terms of absorptivity, distribution, and metabolism as well as its polypharmacological properties need to be studied extensively. The information obtained from the studies conducted on the chemistry of mitragynine **(1)** and its diastereomers could lead toward an efficient botanical extract development of *M. speciosa* where it can be used as an alternative botanical drug in treating pain. Based on this review, the previous preclinical and clinical studies conducted on the mitragynine **(1)** and its diastereomers could support and provide an in-depth insight on the medicinal benefits of this plant, which could lead to drug development in treating pain and addiction. Therefore, it serves as an important research point to prompt medicinal chemists and pharmacologists to conduct extensive studies on the chemistry and synergism activity of mitragynine **(1)** and its diastereomers to understand the opioid-like mechanistic activity relating to the medicinal benefits of this plant.

## References

[B1] Abdullah N. H. Ismail S. (2018). Inhibition of UGT2B7 Enzyme Activity in Human and Rat Liver Microsomes by Herbal Constituents. Molecules 23 (10), 2696. 10.3390/molecules23102696 PMC6222696 30347696

[B2] Basiliere S. Kerrigan S. (2020). Identification of Metabolites and Potential Biomarkers of Kratom in Urine. J. Chromatogr. B Analyt Technol. Biomed. Life Sci. 1140, 121971. 10.1016/j.jchromb.2020.121971 32058315

[B3] Beckett A. H. Shellard E. J. Phillipson J. D. Lee C. M. (1965). Alkaloids from *Mitragyna Speciosa* (Korth.). J. Pharm. Pharmacol. 17, 753–755. 10.1111/j.2042-7158.1965.tb07599.x 4379809

[B4] Berthold E. C. Kamble S. H. Raju K. S. King T. I. Popa R. Sharma A. (2021). Preclinical Pharmacokinetic Study of Speciociliatine, a Kratom Alkaloid, in Rats Using an UPLC-MS/MS Method. J. Pharm. Biomed. Anal. 194, 113778. 10.1016/j.jpba.2020.113778 33277117

[B5] Boffa L. Ghè C. Barge A. Muccioli G. Cravotto G. (2018). Alkaloid Profiles and Activity in Different *Mitragyna Speciosa* Strains. Nat. Prod. Comm. 13 (9). 10.1177/1934578x1801300904

[B6] Brown P. N. Lund J. A. Murch S. J. (2017). A Botanical, Phytochemical and Ethnomedicinal Review of the Genus Mitragyna Korth: Implications for Products Sold as Kratom. J. Ethnopharmacol. 202, 302–325. 10.1016/j.jep.2017.03.020 28330725

[B7] Chakraborty S. DiBerto J. F. Faouzi A. Bernhard S. M. Gutridge A. M. Ramsey S. (2021c). A Novel Mitragynine Analog with Low-Efficacy Mu Opioid Receptor Agonism Displays Antinociception with Attenuated Adverse Effects. J. Med. Chem. 64 (18), 13873–13892. 10.1021/acs.jmedchem.1c01273 34505767 PMC8530377

[B8] Chakraborty S. Majumdar S. (2020). Natural Products for the Treatment of Pain: Chemistry and Pharmacology of Salvinorin A, Mitragynine, and Collybolide. Biochemistry 60 (18), 1381–1400. 10.1021/acs.biochem.0c00629 32930582 PMC7982354

[B9] Chakraborty S. Uprety R. Daibani A. E. Rouzic V. L. Hunkele A. Appourchaux K. (2021b). Kratom Alkaloids as Probes for Opioid Receptor Function: Pharmacological Characterization of Minor Indole and Oxindole Alkaloids from Kratom. ACS Chem. Neurosci. 12 (14), 2661–2678. 10.1021/acschemneuro.1c00149 34213886 PMC8328003

[B10] Chakraborty S. Uprety R. Slocum S. T. Irie T. Le Rouzic V. Li X. (2021a). Oxidative Metabolism as a Modulator of Kratom's Biological Actions. J. Med. Chem. 64 (22), 16553–16572. 10.1021/acs.jmedchem.1c01111 34783240 PMC8673317

[B11] Chear N. J. León F. Sharma A. Kanumuri S. R. R. Zwolinski G. Abboud K. A. (2021). Exploring the Chemistry of Alkaloids from Malaysian *Mitragyna Speciosa* (Kratom) and the Role of Oxindoles on Human Opioid Receptors. J. Nat. Prod. 84 (4), 1034–1043. 10.1021/acs.jnatprod.0c01055 33635670 PMC8693998

[B12] Damodaran T. Chear N. J. Murugaiyah V. Mordi M. N. Ramanathan S. (2021). Comparative Toxicity Assessment of Kratom Decoction, Mitragynine and Speciociliatine versus Morphine on Zebrafish (*Danio rerio*) Embryos. Front. Pharmacol. 12, 714918. 10.3389/fphar.2021.714918 34489704 PMC8417521

[B13] Domnic G. Chear N. J. Y. Rahman S. F. A. Ramanathan S. Lo K. W. Singh D. (2021). Combinations of Indole Based Alkaloids from *Mitragyna Speciosa* (Kratom) and Cisplatin Inhibit Cell Proliferation and Migration of Nasopharyngeal Carcinoma Cell Lines. J. Ethnopharmacol 279, 114391. 10.1016/j.jep.2021.114391 34224811

[B14] Ellis C. R. Racz R. Kruhlak N. L. Kim M. T. Zakharov A. V. Southall N. (2020). Evaluating Kratom Alkaloids Using PHASE. PloS one 15 (3), e0229646. 10.1371/journal.pone.0229646 32126112 PMC7053747

[B15] Flores-Bocanegra L. Raja H. A. Graf T. N. Augustinović M. Wallace E. D. Hematian S. (2020). The Chemistry of Kratom [Mitragyna Speciosa]: Updated Characterization Data and Methods to Elucidate Indole and Oxindole Alkaloids. J. Nat. Prod. 83 (7), 2165–2177. 10.1021/acs.jnatprod.0c00257 32597657 PMC7718854

[B16] Gogineni V. Leon F. Avery B. A. McCurdy C. Cutler S. J. (2015). “Phytochemistry of Mitragyna Speciosa,” in Kratom and Other Mitragynines: Thechemistry and Pharmacology of Opioids from a Non-opium Source. Editor Raffa R. B. (Florida, US: CRC Press), 77–94.

[B17] Goh Y. S. Karunakaran T. Murugaiyah V. Santhanam R. Abu Bakar M. H. Ramanathan S. (2021). Accelerated Solvent Extractions (ASE) of *Mitragyna Speciosa* Korth. (Kratom) Leaves: Evaluation of its Cytotoxicity and Antinociceptive Activity. Molecules 26 (12), 3704. 10.3390/molecules26123704 34204457 PMC8234130

[B18] Hanapi N. A. Chear N. J. Azizi J. Yusof S. R. (2021). Kratom Alkaloids: Interactions with Enzymes, Receptors, and Cellular Barriers. Front. Pharmacol. 12, 751656. 10.3389/fphar.2021.751656 34867362 PMC8637859

[B19] Ilmie M. U. Jaafar H. Mansor S. M. Abdullah J. M. (2015). Subchronic Toxicity Study of Standardized Methanolic Extract of Mitragyna Speciosa Korth in Sprague-Dawley Rats. Front. Neurosci. 9, 189. 10.3389/fnins.2015.00189 26136645 PMC4470260

[B20] Jumali S. S. Said I. M. Ismail I. Zainal Z. (2011). Genes Induced by High Concentration of Salicylic Acid in *Mitragyna Speciosa* . Aust. J. Crop Sci. 5, 296–303.

[B21] Kamble S. H. Sharma A. King T. I. Berthold E. C. León F. Meyer P. K. L. (2020). Exploration of Cytochrome P450 Inhibition Mediated Drug-Drug Interaction Potential of Kratom Alkaloids. Toxicol. Lett. 319, 148–154. 10.1016/j.toxlet.2019.11.005 31707106 PMC7902086

[B22] Kruegel A. C. Gassaway M. M. Kapoor A. Váradi A. Majumdar S. Filizola M. (2016). Synthetic and Receptor Signaling Explorations of the Mitragyna Alkaloids: Mitragynine as an Atypical Molecular Framework for Opioid Receptor Modulators. J. Am. Chem. Soc. 138 (21), 6754–6764. 10.1021/jacs.6b00360 27192616 PMC5189718

[B23] Kruegel A. C. Grundmann O. (2018). The Medicinal Chemistry and Neuropharmacology of Kratom: a Preliminary Discussion of a Promising Medicinal Plant and Analysis of its Potential for Abuse. Neuropharmacology 134, 108–120. 10.1016/j.neuropharm.2017.08.026 28830758

[B24] Kruegel A. C. Uprety R. Grinnell S. G. Langreck C. Pekarskaya E. A. Le Rouzic V. (2019). 7-Hydroxymitragynine Is an Active Metabolite of Mitragynine and a Key Mediator of its Analgesic Effects. ACS Cent. Sci. 5 (6), 992–1001. 10.1021/acscentsci.9b00141 31263758 PMC6598159

[B25] León F. Obeng S. Mottinelli M. Chen Y. King T. I. Berthold E. C. (2021). Activity of *Mitragyna Speciosa* ("Kratom") Alkaloids at Serotonin Receptors. J. Med. Chem. 64 (18), 13510–13523. 10.1021/acs.jmedchem.1c00726 34467758 PMC9235362

[B26] Lu J. Wei H. Wu J. Jamil M. F. Tan M. L. Adenan M. I. (2014). Evaluation of the Cardiotoxicity of Mitragynine and its Analogues Using Human Induced Pluripotent Stem Cell-Derived Cardiomyocytes. PLoS One 9 (12), e115648. 10.1371/journal.pone.0115648 25535742 PMC4275233

[B27] Macko E. Weisbach J. A. Douglas B. (1972). Some Observations on the Pharmacology of Mitragynine. Arch. Int. Pharmacodyn. Ther. 198 (1), 145–161. 4626477

[B28] Matsumoto K. Mizowaki M. Suchitra T. Takayama H. Sakai S. Aimi N. (1996). Antinociceptive Action of Mitragynine in Mice: Evidence for the Involvement of Supraspinal Opioid Receptors. Life Sci. 59 (14), 1149–1155. 10.1016/0024-3205(96)00432-8 8831802

[B29] Matsumoto K. Mizowaki M. Takayama H. Sakai S. Aimi N. Watanabe H. (1997). Suppressive Effect of Mitragynine on the 5-Methoxy-N,n-Dimethyltryptamine-Induced Head-Twitch Response in Mice. Pharmacol. Biochem. Behav. 57 (1-2), 319–323. 10.1016/s0091-3057(96)00314-0 9164589

[B30] Meireles V. Rosado T. Barroso M. Soares S. Gonçalves J. Luís Â. (2019). *Mitragyna Speciosa*: Clinical, Toxicological Aspects and Analysis in Biological and Non-biological Samples. Medicines (Basel) 6 (1), 35. 10.3390/medicines6010035 PMC6473843 30836609

[B31] Nickolls S. A. Waterfield A. Williams R. E. Kinloch R. A. (2011). Understanding the Effect of Different Assay Formats on Agonist Parameters: a Study Using the Μ-Opioid Receptor. J. Biomol. Screen. 16 (7), 706–716. 10.1177/1087057111406548 21550962

[B32] Obeng S. Kamble S. H. Reeves M. E. Restrepo L. F. Patel A. Behnke M. (2020). Investigation of the Adrenergic and Opioid Binding Affinities, Metabolic Stability, Plasma Protein Binding Properties, and Functional Effects of Selected Indole-Based Kratom Alkaloids. J. Med. Chem. 63 (1), 433–439. 10.1021/acs.jmedchem.9b01465 31834797 PMC7676998

[B33] Obeng S. Wilkerson J. L. León F. Reeves M. E. Restrepo L. F. Gamez-Jimenez L. R. (2021). Pharmacological Comparison of Mitragynine and 7-Hydroxymitragynine: *In Vitro* Affinity and Efficacy for μ-Opioid Receptor and Opioid-like Behavioral Effects in Rats. J. Pharmacol. Exp. Ther. 376 (3), 410–427. 10.1124/jpet.120.000189 33384303 PMC7923387

[B34] Oliveira A. S. Fraga S. Carvalho F. Araújo A. M. Pereira C. C. Teixeira J. P. (2016). Chemical Characterization and *In Vitro* Cyto- and Genotoxicity of 'legal High' Products Containing Kratom (Mitragyna Speciosa). Forensic Toxicol. 34 (2), 213–226. 10.1007/s11419-015-0305-6

[B35] Papsun D. M. Chan-Hosokawa A. Friederich L. Brower J. Graf K. Logan B. (2019). The Trouble with Kratom: Analytical and Interpretative Issues Involving Mitragynine. J. Anal. Toxicol. 43 (8), 615–629. 10.1093/jat/bkz064 31424079

[B36] Pearson B. J. Campbell S. M. Avery B. McCurdy C. Francisco J. Sharma A. (2018). Preliminary Examination of Mitragynine and 7-hydroxymitragynine Synthesis in Response to Production Environment and Postharvest Techniques of *Mitragyna Speciosa* . Int. Symp. Beverage Crops. 1274, 89–96.

[B37] Philipp A. A. Wissenbach D. K. Weber A. A. Zapp J. Maurer H. H. (2010). Phase I and II Metabolites of Speciogynine, a Diastereomer of the Main Kratom Alkaloid Mitragynine, Identified in Rat and Human Urine by Liquid Chromatography Coupled to Low- and High-Resolution Linear Ion Trap Mass Spectrometry. J. Mass. Spectrom. 45 (11), 1344–1357. 10.1002/jms.1848 20967737

[B38] Philipp A. A. Wissenbach D. K. Zoerntlein S. W. Klein O. N. Kanogsunthornrat J. Maurer H. H. (2009). Studies on the Metabolism of Mitragynine, the Main Alkaloid of the Herbal Drug Kratom, in Rat and Human Urine Using Liquid Chromatography-Linear Ion Trap Mass Spectrometry. J. Mass. Spectrom. 44 (8), 1249–1261. 10.1002/jms.1607 19536806

[B39] Raffa R. B. (2015). Kratom and Other Mitragynines: The Chemistry and Pharmacologyof Opioids from a Non-opium Source. Florida, US: CRC Press.

[B40] Rosales P. F. Bordin G. S. Gower A. E. Moura S. (2020). Indole Alkaloids: 2012 until Now, Highlighting the New Chemical Structures and Biological Activities. Fitoterapia 143, 104558. 10.1016/j.fitote.2020.104558 32198108

[B41] Sabetghadam A. Ramanathan S. Sasidharan S. Mansor S. M. (2013). Subchronic Exposure to Mitragynine, the Principal Alkaloid of *Mitragyna Speciosa*, in Rats. J. Ethnopharmacol. 146 (3), 815–823. 10.1016/j.jep.2013.02.008 23422336

[B42] Saidin N. A. Holmes E. Takayama H. Gooderham N. J. (2015). The Cellular Toxicology of Mitragynine, the Dominant Alkaloid of the Narcotic-like Herb, *Mitragyna Speciosa* Korth. Toxicol. Res. 4 (5), 1173–1183. 10.1039/c5tx00113g

[B43] Shellard E. Houghton P. Resha M. (1978). The Mitragyna Species of Asia. Planta Med. 34 (07), 253–263. 10.1055/s-0028-1097448 4261718

[B44] Takayama H. (2004). Chemistry and Pharmacology of Analgesic Indole Alkaloids from the Rubiaceous Plant, *Mitragyna Speciosa* . Chem. Pharm. Bull. (Tokyo) 52 (8), 916–928. 10.1248/cpb.52.916 15304982

[B45] Takayama H. Ishikawa H. Kurihara M. Kitajima M. Aimi N. Ponglux D. (2002). Studies on the Synthesis and Opioid Agonistic Activities of Mitragynine-Related Indole Alkaloids: Discovery of Opioid Agonists Structurally Different from Other Opioid Ligands. J. Med. Chem. 45 (9), 1949–1956. 10.1021/jm010576e 11960505

[B46] Veltri C. Grundmann O. (2019). Current Perspectives on the Impact of Kratom Use. Subst. Abuse Rehabil. 10, 23–31. 10.2147/SAR.S164261 31308789 PMC6612999

[B47] Wilson L. L. Harris H. M. Eans S. O. Brice-Tutt A. C. Cirino T. J. Stacy H. M. (2020). Lyophilized Kratom tea as a Therapeutic Option for Opioid Dependence. Drug Alcohol Depend 216, 108310. 10.1016/j.drugalcdep.2020.108310 33017752

[B48] Ya K. Tangamornsuksan W. Scholfield C. N. Methaneethorn J. Lohitnavy M. (2019). Pharmacokinetics of Mitragynine, a Major Analgesic Alkaloid in Kratom (*Mitragyna Speciosa*): A Systematic Review. Asian J. Psychiatr. 43, 73–82. 10.1016/j.ajp.2019.05.016 31100603

[B49] Zacharias D. E. Rosenstein R. D. Jeffrey G. A. (1965). The Structure of Mitragynine Hydroiodide. Acta Cryst. 18 (6), 1039–1043. 10.1107/s0365110x65002499

[B50] Zhou Y. Ramsey S. Provasi D. El Daibani A. Appourchaux K. Chakraborty S. (2021). Predicted Mode of Binding to and Allosteric Modulation of the μ-Opioid Receptor by Kratom's Alkaloids with Reported Antinociception *In Vivo* . Biochemistry 60 (18), 1420–1429. 10.1021/acs.biochem.0c00658 33274929 PMC8119294

